# Surgical Explantation of a Balloon-Expandable Transcatheter Aortic Valve With Aortic Root Preservation for Prosthetic Valve Endocarditis: A Case Report

**DOI:** 10.7759/cureus.115851

**Published:** 2026-09-06

**Authors:** Victor Manuel Carmona Delgado, Jose Emanuel Pereyda Gamez, Diego J Oregel Camacho, Jennifer Zamudio Lizarraga, Ana Alejandra Morales González

**Affiliations:** 1 Cardiothoracic Surgery, Centro Medico Nacional Siglo XXI, Ciudad de México, MEX

**Keywords:** aortic endocarditis, endovascular endocarditis, enterococcus faecalis (e. faecalis), tavi complications, transarterial aortic valve implantation (tavi)

## Abstract

Infective endocarditis following transcatheter aortic valve implantation represents an increasingly recognized and devastating clinical entity characterized by severe morbidity, high mortality, and formidable surgical complexity. As indications for transcatheter aortic valve procedures expand toward younger and lower-risk patient cohorts, late-onset endocarditis poses significant therapeutic dilemmas. Surgical management is technically challenging due to dense tissue integration, neointimal proliferation, and complete endothelialization of the expanded metallic frame into the aortic wall and subvalvular structures. We report the case of a 77-year-old male with a history of transfemoral transcatheter aortic valve implantation (TAVI) and permanent dual-chamber pacemaker placement who presented with late-onset *Enterococcus faecalis* infective endocarditis. The patient developed severe prosthetic valve dysfunction alongside secondary severe functional mitral regurgitation. Comprehensive multiplanar and three-dimensional transesophageal echocardiography identified extensive mobile vegetations significantly obstructing the prosthetic lumen. The patient successfully underwent urgent open cardiac surgery, consisting of complete explantation of the heavily endothelialized metallic stent frame via meticulous peri-stent sharp dissection, biological aortic valve replacement with aortic root preservation, and ring mitral annuloplasty. The postoperative course was uneventful, and the patient completed six weeks of targeted intravenous antimicrobial therapy with clinical and echocardiographic improvement. Surgical explantation with preservation of the native aortic root may represent a feasible therapeutic strategy in selected patients with TAVI-associated infective endocarditis requiring surgical intervention, particularly in the presence of severe prosthetic valve dysfunction, extensive vegetation burden, or associated multivalvular disease.

## Introduction

Over the past decade, transcatheter aortic valve implantation (TAVI) has revolutionized the management of symptomatic severe aortic stenosis. Initially reserved for inoperable or high-surgical-risk candidates, TAVI has demonstrated non-inferiority and favorable outcomes compared with conventional surgical aortic valve replacement (SAVR), leading to its progressive expansion into intermediate- and low-risk patient cohorts [1,2]. As the global volume of TAVI procedures increases and the technology is increasingly applied to patients with longer life expectancies, late procedural complications have emerged as important clinical concerns [3,4].

Infective endocarditis involving a TAVI bioprosthesis (TAVI-IE) represents an uncommon but potentially severe post-procedural complication, with reported incidence ranging from approximately 0.5% to 3.1% [5,6]. TAVI-IE presents with clinical and microbiological characteristics that may differ from those of native valve endocarditis (NVE) and conventional surgical prosthetic valve endocarditis (PVE) [7]. Patients undergoing TAVI are often elderly and have multiple comorbidities, frailty, and frequent healthcare exposure, which may increase their susceptibility to bloodstream infection [5-7]. The presence of a transcatheter prosthetic valve provides an additional substrate for prosthetic valve infection, and TAVI-associated infective endocarditis has been associated with substantial morbidity and mortality [5,6,8]. *Enterococcus faecalis *and *Staphylococcus aureus *are among the predominant pathogens isolated in TAVI-IE [5,6].

Surgical intervention in TAVI-IE presents substantial technical challenges that may exceed those encountered during conventional reoperative SAVR [9,10]. Over time, radial forces exerted by balloon-expandable or self-expanding stent frames, together with the host tissue response, may result in tissue integration and neointimal proliferation involving the transcatheter prosthesis and surrounding structures [10,11]. This tissue incorporation can distort normal surgical anatomy and complicate prosthesis explantation, increasing the risk of injury to the native aortic annulus, aortic-mitral continuity, and coronary ostia [10-12].

Although international clinical practice guidelines advocate early surgical intervention in complicated prosthetic valve endocarditis, particularly in the presence of severe prosthetic dysfunction, heart failure, uncontrolled infection, or high embolic risk [13,14], surgical treatment of TAVI-associated infective endocarditis remains challenging. Advanced comorbidities, high operative risk, and the technical complexity of transcatheter prosthesis explantation may limit surgical management in this population [15]. In this report, we describe the successful surgical management of a complex case of late-onset *Enterococcus faecalis *TAVI-associated infective endocarditis with multivalvular involvement through complete transcatheter frame explantation, aortic root preservation, biological aortic valve replacement, and concomitant mitral ring annuloplasty.

## Case presentation

Patient profile and clinical presentation

A 77-year-old male with a medical history of systemic arterial hypertension, type 2 diabetes mellitus, and recurrent urinary tract infections underwent transfemoral TAVI using a 23 mm balloon-expandable bioprosthesis for severe degenerative aortic stenosis. His early post-TAVI clinical course was complicated by complete atrioventricular block, necessitating permanent dual-chamber (DDDR) endocardial pacemaker placement. Given the patient's history of permanent dual-chamber pacemaker placement following the index TAVI procedure, special clinical attention was dedicated to evaluating the cardiac implantable electronic device (CIED) in the setting of *Enterococcus faecalis *bacteremia. Physical examination confirmed a completely normal generator pocket without erythema, local warmth, or tenderness. Furthermore, comprehensive multiplanar and three-dimensional transesophageal echocardiography (TEE) specifically scrutinized the endocardial leads along their entire trajectory, demonstrating no evidence of lead-associated masses or mobile vegetations. Following multidisciplinary Heart Team consultation, device extraction was considered; given the clean pocket presentation, the absence of lead involvement on imaging, and the infection being anatomically localized strictly to the aortic bioprosthesis and secondary mitral apparatus, a conservative strategy retaining the functioning pacemaker system was successfully maintained alongside targeted synergistic antimicrobial therapy.

Four months before surgical evaluation, the patient developed an insidious, intermittent fever of unknown origin (>38.5°C), accompanied by profound fatigue, night sweats, and progressive malaise. He received multiple empirical outpatient oral antimicrobial courses without sustained clinical resolution.

Upon admission to our tertiary cardiovascular referral center, physical examination demonstrated hemodynamic stability with a blood pressure of 115/70 mmHg, a heart rate of 82 beats/min, a respiratory rate of 18 breaths/min, and a grade III/VI harsh systolic ejection murmur at the right upper sternal border radiating to both carotid arteries. Physical examination revealed no peripheral stigmata of endocarditis such as Janeway lesions, Osler nodes, or Roth spots.

Diagnostic workup and laboratory evaluation

Initial laboratory evaluation demonstrated significant inflammatory activation with marked leukocytosis, a left shift, elevated acute-phase reactants, and mild normocytic anemia, while renal function remained within normal parameters. Three independent sets of serial peripheral blood cultures grew *Enterococcus faecalis *susceptible to ampicillin, vancomycin, and high-dose aminoglycosides. The complete laboratory findings at admission, along with institutional reference ranges, are summarized in Table 1.

**Table 1:** Preoperative Laboratory Workup at Admission

Investigation / Marker	Patient Value	Reference Range
White Blood Cell (WBC) Count	14.2 × 10^3 μL (82% neutrophils)	4.5−11.0 × 10^3 μL
C-Reactive Protein (CRP)	128 mg/L	<5.0 mg/L
Procalcitonin (PCT)	3.4 ng/mL	<0.1 ng/mL
Serum Creatinine	1.1 mg/dL	0.7−1.3 mg/dL
Hemoglobin	10.2 g/dL	13.0−17.0 g/dL

Multiplanar and 3D TEE was performed to assess valvular morphology and hemodynamics. Figure 1A demonstrates multiplanar views of the aortic root with large, irregular, mobile vegetations attached to the prosthetic leaflets and inner stent struts. Figure 1B provides a 3D volumetric en-face visualization showing total luminal compromise of the transcatheter bioprosthesis due to massive vegetation burden. Figure 1C shows color Doppler TEE illustrating severe prosthetic dysfunction manifesting as a combined double aortic lesion (peak velocity 4.2 m/s, mean gradient 44 mmHg), with moderate intra-prosthetic regurgitation alongside secondary severe functional mitral regurgitation caused by posterior annular dilatation and spatial distortion from adjacent tissue inflammation. Left ventricular systolic function was preserved with an ejection fraction (LVEF) of 56%.

**Figure 1:**
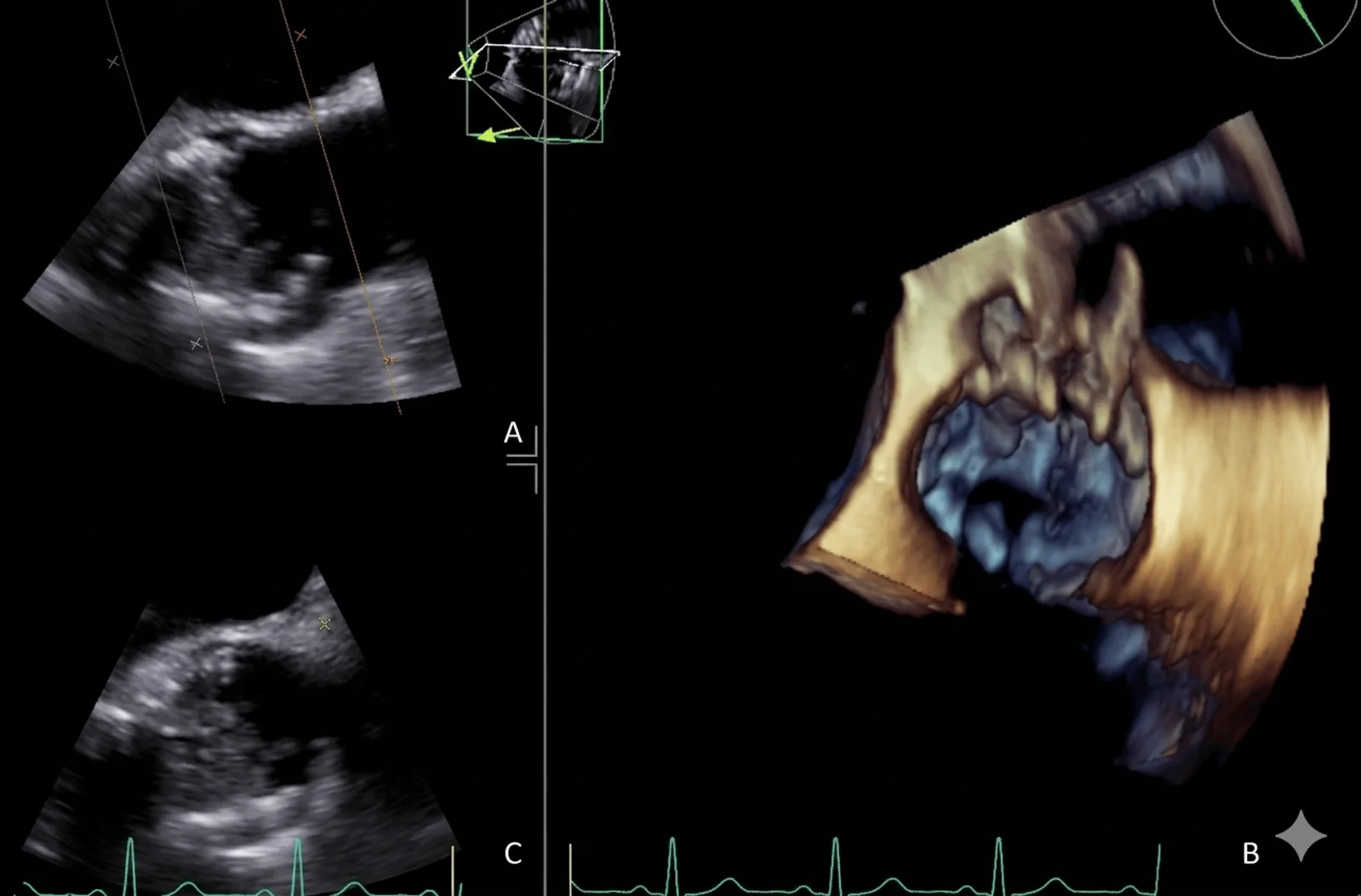
Preoperative Transesophageal Echocardiogram Showing TAVI Endocarditis Multiplanar and 3D transesophageal echocardiography (TEE) demonstrating a transcatheter aortic valve bioprosthesis with extensive, mobile, irregular vegetations adhered to the prosthetic leaflets and inner stent frame. (A) Multiplanar 2D view showing large vegetation burden. (B) 3D volumetric view showing luminal narrowing by vegetation mass. (C) Color Doppler showing combined double aortic lesion and severe secondary mitral regurgitation. TEE: transesophageal echocardiography; TAVI: transcatheter aortic valve implantation.

The diagnosis of definite prosthetic valve infective endocarditis was established according to the 2023 Duke-International Society for Cardiovascular Infectious Diseases (ISCVID) criteria, based on the presence of a major microbiological criterion with multiple blood cultures positive for *Enterococcus faecalis *and a major imaging criterion demonstrating prosthetic valve involvement with large vegetations on transesophageal echocardiography [14]. Targeted intravenous antimicrobial therapy with ampicillin (2 g IV every 4 hours) plus ceftriaxone (2 g IV every 12 hours) was initiated. Given the combination of severe prosthetic valve dysfunction, secondary severe mitral regurgitation, extensive vegetation burden, and high embolic potential, the multidisciplinary Heart Team recommended urgent surgical intervention.

The indication for urgent surgical intervention was based on the combination of active *Enterococcus faecalis *prosthetic valve infection, severe transcatheter valve dysfunction, extensive prosthetic valve vegetations, and severe secondary mitral regurgitation. The prosthetic valve demonstrated a mean transvalvular gradient of 44 mmHg with associated intraprosthetic regurgitation, consistent with significant mechanical obstruction and dysfunction. The extensive vegetation burden, with near-complete luminal compromise on three-dimensional transesophageal echocardiography, further increased the risk of systemic embolization. Given the severity of the prosthetic dysfunction, the extent of the infected prosthetic material, and the associated multivalvular disease, the Heart Team considered surgical source control necessary rather than continued medical therapy alone.

Under general balanced anesthesia, invasive arterial line monitoring, and continuous TEE guidance, a conventional median sternotomy was performed. Cardiopulmonary bypass (CPB) was established using ascending aortic cannulation and bicaval venous cannulation under moderate hypothermia (32 °C). Following aortic cross-clamping, myocardial protection was achieved via initial anterograde cold blood cardioplegia into the aortic root, supplemented by retrograde delivery through the coronary sinus every 20 minutes. A transverse aortotomy was performed approximately 1.5 cm superior to the sinotubular junction. Direct intraoperative inspection revealed the 23 mm prosthesis anchored tightly to the sinuses of Valsalva and native aortic annulus, heavily encrusted with friable vegetations and covered by dense neointimal tissue along its outer perimeter.

To avoid native aortic root avulsion, a meticulous circumferential dissection was performed using a #11 scalpel blade and fine Metzenbaum scissors to develop the fibrotic cleavage plane between the outer expanded metallic stent struts and the native aortic wall. Gentle, controlled inward traction was applied to collapse the frame inward without tearing the native fibrous skeleton or injuring the coronary ostia, allowing complete, intact removal of the transcatheter prosthesis without disruption of the aortic-mitral continuity (Figure 2). Following radical debridement of all infected and devitalized tissue, the left ventricular outflow tract and aortic root were copiously irrigated with warm dilute povidone-iodine and saline solution. Because the native annulus remained structurally sound, a 23 mm biological aortic valve was implanted using interrupted 2-0 polyester pledgeted sutures placed in a supra-annular configuration, ensuring appropriate seating and complete coronary ostial patency without requiring pericardial patch reconstruction.

**Figure 2:**
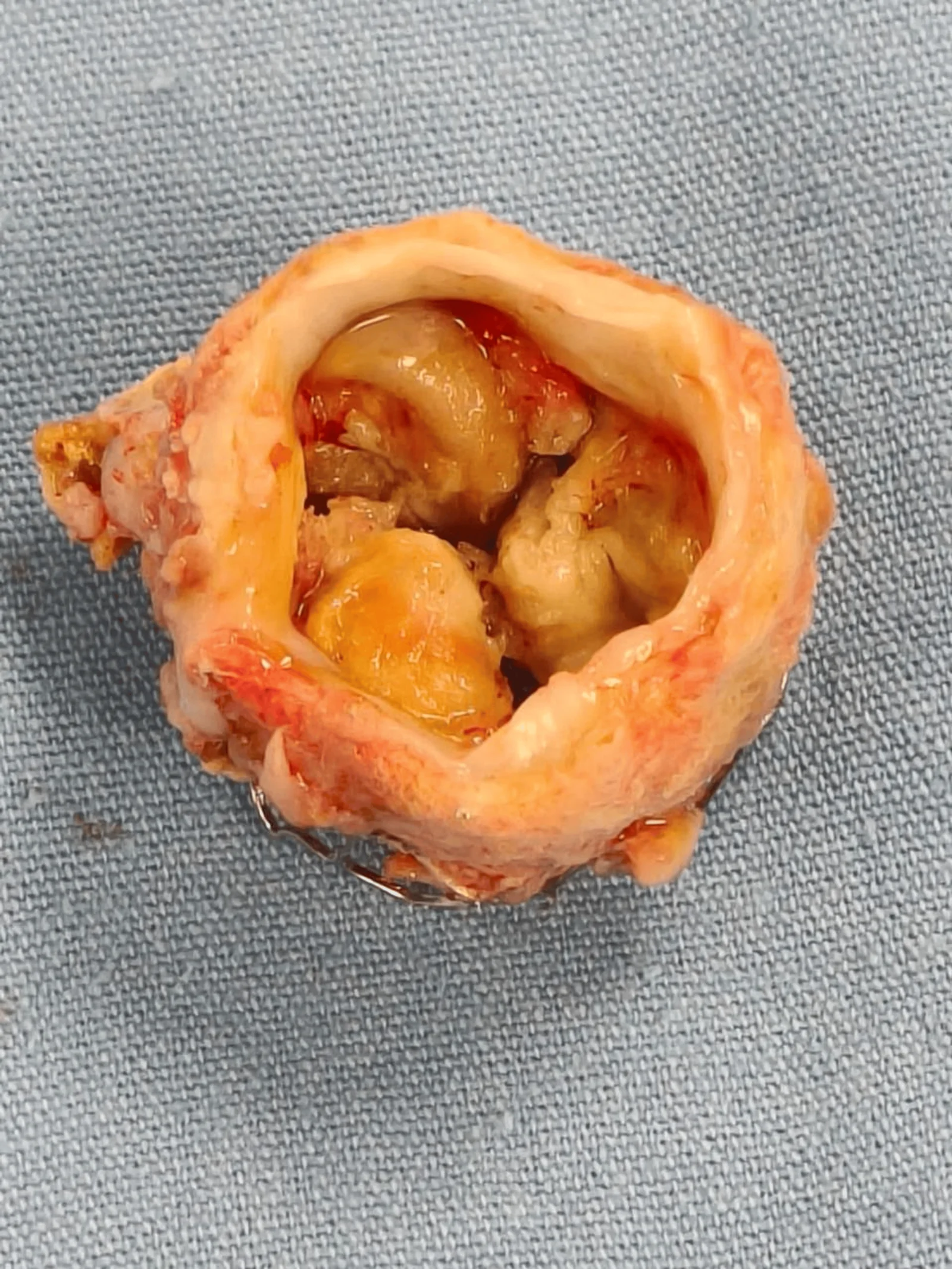
Gross Pathological Specimen of the Explanted Transcatheter Valve Surgical gross specimen of the explanted 23 mm transcatheter aortic valve bioprosthesis following complete excision. The image shows the intact metallic frame lined with significant circumferential neointimal tissue/fibrosis along its outer perimeter, and large, friable, yellowish-green bacterial vegetations virtually obliterating the prosthetic leaflets and inner lumen.

Access to the mitral valve was subsequently gained via a standard left atriotomy along the interatrial groove (Sondergaard's groove). Inspection confirmed that the severe mitral regurgitation was secondary to posterior annular dilatation with poor leaflet coaptation, in the absence of leaflet destruction or chordal rupture. A ring annuloplasty was successfully performed using a 25 mm complete semirigid ring, and saline hydraulic testing demonstrated complete leaflet competence with a broad zone of coaptation and preservation of the aortic-mitral continuity. Total CPB time was 153 minutes, and aortic cross-clamp time was 130 minutes. The patient was successfully weaned from CPB in sinus rhythm without inotropic requirement or conduction abnormalities.

Postoperative course

Following tertiary hospital admission and rapid hemodynamic stabilization, urgent surgical intervention was successfully performed within 48 hours of admission. Following surgical explantation, microbiological cultures and histopathological examination of the extracted transcatheter aortic valve prosthesis and associated vegetations were performed, confirming tissue involvement and inflammatory infiltration by *Enterococcus faecalis*. Additionally, serial blood cultures were monitored, documenting successful microbiological clearance following the initiation of targeted antimicrobial therapy.

The patient was transferred to the Cardiovascular Surgical Intensive Care Unit under low-dose vasoactive support, which was completely weaned within 36 hours. Early extubation was achieved at 8 hours postoperatively. Serial postoperative laboratory tests showed rapid normalization of inflammatory markers, with the white blood cell count decreasing to 7.1 x 10^3 μL (reference range: 4.5 - 11.0 x 10^3 μL) and C-reactive protein falling to 12 mg/L at two weeks post-surgery.

Postoperative control TEE confirmed a well-functioning aortic bioprosthesis with normal transvalvular gradients (mean gradient 9 mmHg, peak velocity 2.1 m/s), absence of paravalvular leak, a fully competent mitral valve repair without residual regurgitation, and a preserved LVEF of 58%.

The patient completed a six-week regimen of targeted intravenous antibiotic therapy with ampicillin (2 g IV every 4 h) plus ceftriaxone (2 g IV every 12 h) [13,14]. His inpatient recovery was uncomplicated, free of neurological events, vascular complications, or infectious recurrence. He was discharged home on postoperative day 28 for structured outpatient surveillance. At 12-month clinical follow-up, the patient remains asymptomatic in New York Heart Association (NYHA) Functional Class I with stable valvular hemodynamics.

## Discussion

The clinical chronology unfolded as follows: The patient underwent transfemoral TAVI, followed shortly by permanent pacemaker implantation due to complete atrioventricular block. Approximately 36 months following the index procedure (representing late-onset prosthetic valve endocarditis), the patient developed an insidious, 4-month history of intermittent fever of unknown origin, receiving empirical outpatient therapies before tertiary center admission. Upon arrival, serial blood cultures yielded *Enterococcus faecalis *within 48 hours, prompting immediate targeted antimicrobial therapy and urgent surgical intervention within the same hospitalization. Postoperatively, microbiological clearance and a 6-week intravenous antibiotic regimen were successfully completed, culminating in an uneventful 12-month follow-up showing New York Heart Association (NYHA) Functional Class I status. Regarding the microbiological source of infection, given the patient's documented medical history of recurrent urinary tract infections, a detailed urological assessment and baseline urine cultures were explicitly integrated into the clinical evaluation, identifying the genitourinary tract as the most probable primary portal of entry.

Infective endocarditis following transcatheter aortic valve implantation represents one of the most challenging scenarios encountered in adult cardiac surgery [5,9]. While TAVI has dramatically reduced morbidity and mortality in patients with severe aortic stenosis, TAVI-associated infective endocarditis is associated with substantial mortality, with reported in-hospital mortality of approximately 36-47% and one-year mortality approaching 66% in large observational series [5,6]

Pathophysiology and microbiological drivers

The pathophysiology of TAVI-IE differs fundamentally from native valve endocarditis [8]. The expansion of a balloon-expandable or self-expanding metal stent frame causes focal micro-trauma to the native aortic wall, leaving exposed metallic struts that serve as a nidus for bacterial adhesion and dense biofilm production [8,10]. *Enterococcus faecalis *is consistently identified as the second or third most common causative pathogen in TAVI-IE globally, accounting for 20-30% of cases [5,6]. Enterococcal bacteremia frequently originates from gastrointestinal or genitourinary portals of entry-common sites of routine healthcare interventions in elderly, frail candidates [7,16]. Compared to streptococcal endocarditis, *E. faecalis *PVE exhibits a higher frequency of destructive invasive features, larger vegetation burden, and resistance to standard monotherapy, requiring prolonged synergistic bactericidal antibiotic regimens such as ampicillin plus ceftriaxone [13,14,16].

Technical challenges of TAVI explantation and the EXPLANT-TAVR context

Data from the international EXPLANT-TAVR registry and the Society of Thoracic Surgeons (STS) database indicate that surgical explantation following transcatheter aortic valve failure is associated with significant operative mortality (ranging from 13% to 28%), driven largely by patient frailty, emergency status, and technical complexity [9,15,17]. Bapat et al. and Fukuhara et al. emphasized that complete excision of the transcatheter stent frame requires establishing a clear anatomical plane between the outer metallic struts and the aortic wall using sharp scalpel dissection [9,10]. Specifically, in balloon-expandable devices, once circumferential sharp dissection frees the upper crown, applying gentle inward radial traction with non-toothed forceps allows controlled in situ crimping and mechanical collapse of the 23 mm cobalt-chromium frame away from the native aortic wall [10,12]. This maneuver may facilitate controlled explantation while reducing the risk of aortic root injury and the need for complex root reconstruction [11,12]. In our case, strict adherence to this standardized peri-stent dissection and inward collapsing strategy allowed complete removal of the infected prosthesis while preserving native annular structural integrity [10,12].

Multivalvular involvement and surgical decision-making

Secondary involvement of the mitral valve in aortic PVE can occur via direct contiguous extension through the aortic-mitral continuity, vegetation drop/seeding, or secondary annular dilatation from acute volume overload [18]. When feasible, mitral valve repair may avoid implantation of a second prosthesis and has been associated with favorable long-term outcomes compared with double valve replacement in selected patients undergoing combined aortic and mitral surgery [18]. In the present case, meticulous intraoperative inspection revealed that the severe mitral regurgitation was entirely functional, resulting from posterior annular dilation without structural leaflet destruction or abscess formation in the aortic-mitral continuity. Performing a ring annuloplasty preserved native LV-arterial coupling, minimized cross-clamp time (130 minutes), and restored complete valvular competence without the added risk of a second prosthetic implant.

Epidemiological and surgical context in Mexico

In Mexico, the use of transcatheter aortic valve implantation has progressively expanded since the introduction of the technique into specialized cardiovascular centers. The first reported Mexican experience with a balloon-expandable transcatheter valve was published in 2014, marking the beginning of a local TAVI program and demonstrating the feasibility of this technology in a Mexican tertiary cardiovascular setting [19]. As TAVI utilization has increased, prosthetic valve-related complications, including infective endocarditis, have become increasingly relevant clinical challenges. More recently, a Mexican case report described successful surgical aortic valve replacement following infective endocarditis involving a transcatheter aortic valve, highlighting the feasibility of surgical conversion in selected patients with TAVR-associated complications [20]. Nevertheless, published Mexican experience regarding the surgical management of TAVI-associated infective endocarditis remains limited. Our case adds to the existing literature by providing detailed surgical and echocardiographic documentation of complete explantation of an infected balloon-expandable TAVI prosthesis with preservation of the native aortic root and concomitant mitral valve repair.

## Conclusions

Surgical explantation of an infected TAVI prosthesis combined with biological aortic valve replacement and concomitant mitral valve repair may be a feasible approach in selected patients with prosthetic valve endocarditis requiring surgical intervention. Meticulous circumferential peri-stent dissection along the tissue-frame interface may facilitate complete prosthesis removal while preserving the native aortic root when adequate tissue planes and annular integrity are present.

Early multidisciplinary evaluation by a dedicated Heart Team is important to identify appropriate surgical candidates before progression to severe hemodynamic compromise or extensive tissue destruction.
